# Preventive Intake of a Multiple Micronutrient Supplement during Mild, Acute SARS-CoV-2 Infection to Reduce the Post-Acute COVID-19 Condition: A Double-Blind, Placebo-Controlled, Randomized Clinical Trial

**DOI:** 10.3390/nu16111631

**Published:** 2024-05-26

**Authors:** Teresa Maria Tomasa-Irriguible, Ramon Monfà, Cristina Miranda-Jiménez, Rosa Morros, Neus Robert, Luisa Bordejé-Laguna, Sandra Vidal, Pere Torán-Monserrat, Ana Maria Barriocanal

**Affiliations:** 1 Intensive Care Department, Hospital Universitari Germans Trias i Pujol, 08916 Badalona, Spain; lbordeje.germanstrias@gencat.cat; 2 Jordi Gol University Research Institute in Primary Care (IDIAP Jordi Gol), 08007 Barcelona, Spain; rmonfa@idiapjgol.info (R.M.); cmiranda@idiapjgol.org (C.M.-J.); 3 Department of Pharmacology, Therapeutics and Toxicology, Autonomous University of Barcelona (UAB), 08193 Bellaterra, Spain; rmorros.bnm.ics@gencat.cat; 4 Emergency Department, Hospital Universitari Germans Trias i Pujol, 08916 Badalona, Spain; nrobert.germanstrias@gencat.cat; 5 Germans Trias i Pujol Research Institute (IGTP), 08916 Badalona, Spain; svidal@igtp.cat (S.V.); ambarriocanal@igtp.cat (A.M.B.); 6 North Metropolitan Research Support Unit, Jordi Gol University Research Institute in Primary Care (NM-IDIAP Jordi Gol), 08303 Mataró, Spain; ptoran.bnm.ics@gencat.cat

**Keywords:** COVID-19, SARS-CoV-2, post-COVID-19 condition, long COVID, nutritional status, micronutrients, multiple micronutrient supplement, cognitive assessment, cognitive impairment, quality of life

## Abstract

Patients hospitalized with COVID-19 have low levels of vitamins and trace elements. This could lead to a post-acute COVID-19 condition (PCC) that can worsen a patient’s quality of life. We aimed to study the baseline micronutrient status of patients and assess whether a multiple micronutrient supplement (MMS) taken for 2 weeks at the first sign of COVID-19 symptoms would be able to reduce the incidence of PCC. This double-blind, placebo-controlled, randomized clinical trial was conducted in adult outpatients with acute COVID-19, recruited between 2021 and 2023 in Spain. Of the 285 patients assessed for eligibility, 267 were randomized and 246 were included in the intent-to-treat population. The mean age was 46.8 years, and 68% were female. Overall, 54.6% had micronutrient deficiencies in the acute phase of COVID-19 at baseline, and 26.2% had PCC after 180 days of follow-up (D180). The most frequently recorded PCC symptoms were neurological (14.1%), with 24% patients scoring worse in the cognitive tests compared to their baseline status. The rate of PCC at D180 was similar between the placebo (25.0%) and intervention (27.7%) groups, without significant differences (*p* = 0.785). Age over 50 years was the most relevant risk factor for developing PCC, followed by female sex. The most important protective factor against PCC was SARS-CoV-2 vaccination. In this population of predominantly middle-aged, white women with acute COVID-19 not requiring hospital admission, MMS intake for 14 days at symptom onset did not prevent PCC nor improve their micronutrient status at D180.

## 1. Introduction

The COVID-19 pandemic has caused millions of deaths and hospitalizations and elicited a high socioeconomic cost. With the pandemic over, and despite all the scientific advances in the treatment and prevention of the disease, SARS-CoV-2 continues to cause health problems and may lead to persistent symptoms known as the post-acute COVID-19 condition (PCC), which can impair the quality of life of these patients [1,2,3]. 

To date, it is still unknown why some patients have severe COVID-19 while others have mild symptoms or even remain asymptomatic, or why some patients develop PCC and others do not. A genetic substrate that predisposes one to the disease has been described, although these discoveries have had limited clinical impact [4]. Nonetheless, other predisposing and perhaps modifiable factors may also contribute to this condition, so it remains necessary to continue studying those factors that may predispose suffering and investigate how to minimize symptoms. 

The role of micronutrients in the prophylaxis of infections in general has been the subject of study for years. A recent pre-pandemic review has appraised the role of micronutrients in the immune response to infections, particularly viral infections [5], highlighting that the micronutrients with the greatest evidence of immunological activity are vitamins C and D and zinc. Vitamins A, E, B6, and B12, folic acid, and minerals such as iron, copper, and selenium also play other roles at each stage of the immune response [6]. 

Throughout the first wave of the COVID-19 pandemic, a Spanish epidemiological study analyzed the micronutritional status of 200 patients hospitalized for COVID-19 [7] and observed that most of these patients had low levels of several micronutrients upon admission, including vitamin C (82%), vitamin D (74%), vitamin A (68%), vitamin B6 (45%), and zinc (70%). Low levels of zinc and vitamin A, age over 65 years, and male sex were found to be associated with greater severity in hospitalized patients. 

As time went by and the pandemic waned—mostly attributable to less lethal variants of the virus, herd immunity, and SARS-CoV-2 vaccination—the need for hospitalization and intensive care radically decreased. The focus changed to the persistence of disease symptoms or long convalescence, an entity that is not yet well defined, and is named differently as persistent COVID, long COVID, or post-acute COVID-19 condition (PCC) [8]. A diversity of persistent symptoms has been described (such as neurological, respiratory, cardiovascular, or digestive effects, fatigue, etc.) that can impair the quality of life of PCC patients. Currently, PCC is a topic of concern for SARS-CoV-2 infection, estimated to have an incidence of up to 20% [9]. PCC can even develop in patients with a mild, acute COVID-19 presentation, another matter of concern [10]. Furthermore, the duration of PCC is uncertain (to date, reportedly lasting for 2 years) and its socioeconomic–health impact has not yet been appraised [11,12,13]. 

Accordingly, we carried out a randomized, double-blind, placebo-controlled clinical trial in outpatients with acute COVID-19, with two main objectives: (1) to assess the incidence of PCC and analyze its association with the baseline micronutrient status, diet, SARS-CoV-2 vaccination status, and underlying diseases; (2) to analyze the effect of multiple micronutrient supplement (MMS) intake during acute SARS-CoV-2 infection to prevent PCC and hospital admission. Cognitive status and quality of life were defined as secondary outcomes. 

## 2. Materials and Methods

### 2.1. Trial Design 

This was a multicenter, two-arm, parallel group, double-blind, randomized clinical trial conducted in two centers in Catalonia, Spain. The recruiting physicians started the study in September 2021, and the study ended in February 2023 when the sample size had been achieved. Written informed consent was obtained from all patients before screening. The study protocol was approved by the Ethics Committee of the Germans Trias i Pujol University Hospital (number PI-20-381). 

Study data were collected and managed using REDCap (Research Electronic Data Capture) tools, hosted at Fundació Institut Universitari per a la recerca a l’Atenció Primària de Salut Jordi Gol i Gurina (IDIAPJGol) [14,15]. REDCap is a secure, web-based software platform designed to support data capture for research studies, providing (1) an intuitive interface for validated data capture; (2) audit trails for tracking data manipulation and export procedures; (3) automated export procedures for seamless data downloads to common statistical packages; and (4) procedures for data integration and interoperability with external sources.

See the full description of trial procedures in the Supplementary Materials (Material S1).

### 2.2. Study Population 

Patients aged 18 years or older with COVID-19 compatible symptoms and a positive SARS-CoV-2 test, but with no need for hospital admission, were screened. None of the following conditions were exclusion criteria and patients presenting any of them could be eligible for inclusion: previous SARS-CoV-2 infection; SARS-CoV-2 vaccination; suspected active viral or bacterial infection; severely immunocompromised people—including subjects with known human immunodeficiency virus (HIV-1) infection; neutropenic subjects with <500 neutrophils/mm^3^; subjects with solid organ transplantation; subjects with bone marrow transplantation; subjects undergoing chemotherapy; subjects with primary immunodeficiency; severe lymphopenia with <400 lymphocytes/mm^3^; treatment with any anti-cytokine therapy; oral treatment with steroids (defined as daily doses of 10 mg of prednisone or equivalent for more than 3 months); and active solid or non-solid malignancy or lymphoma from the previous 2 years.

The exclusion criteria were as follows: intake of one supplement combining multiple micronutrients during the month prior to inclusion; patients fulfilling hospitalization criteria; previous allergies to the micronutrient components and excipients; age ≥90 years with any comorbidity (diabetes, hypertension, obesity, heart disease, respiratory disease); participation in other research that required experimental intervention (did not include observational studies) in the previous month before signing the informed consent form; detection by the researcher of lack of knowledge or willingness to participate and comply with all the requirements of the protocol; any other findings that at the discretion of the researcher may have compromised protocol performance or significantly influenced the interpretation or results of the effects of the nutritional supplement; and pregnancy or breastfeeding.

### 2.3. Micronutrients: Sample Collection and Measurements

Sample collection was performed at baseline and during the follow-up visit on day 90 (D90). The analyses were carried out at the Referral Laboratory (Laboratori Clínic Metropolitana Nord) of Germans Trias i Pujol Hospital. Micronutrient levels were measured in addition to the usual hematology and biochemistry parameters. 

In case the patient needed to be admitted to the hospital, another blood sample extraction was performed for micronutrients within the first 24 h after hospital admission.

Sample extraction was performed by venipuncture (in code bar labeled tubes), with amounts adequate for each parameter. Samples for vitamin determination were quickly brought to the laboratory for immediate processing or adequate storage. Tubes for the determination of unstable parameters were also immediately protected from light (vitamin C denatures with light and temperature and is easily oxidized). 

High-performance liquid chromatography methods were used to measure vitamins A, E, B1, B6, and C. Immunoassay methods were employed to measure vitamin B12, folate, and 25-OH vitamin D. Colorimetric devices measured copper, zinc, and iron, while inductively coupled plasma mass spectrometry measured selenium.

### 2.4. Macronutrients: Recording and Interpretation

Food intake was considered poor if both quantitative and qualitative deficiencies were recorded in the week prior to study inclusion. Quantitative poor dietary intake was considered if the patient had ingested <25% of the usual intake. Poor qualitative food intake was considered when (1) the variability of the macronutrients recorded (e.g., legumes, meat, fish, eggs, vegetables, fruits, cereals, milk) was <3 types, or (2) the groups of nutrients were <2 types: (a) proteins (meat, fish, eggs, legumes, and milk), (b) fruits/juices and vegetables, and (c) cereals.

### 2.5. Interventions: Randomization and Sequence Generation

Study treatment began on day 1 at inclusion and lasted for 14 consecutive days. Both treatments (MMS and placebo) were effervescent tablets with the same external aspect. The components of the MMS (supplied by Bayer Consumer Care, Basel, Switzerland) were as follows: retinol (Vitamin A) 700 mcg; cholecalciferol (vitamin D3) 10 mcg; alpha-tocopherol (vitamin E) 45 mg; ascorbic acid (vitamin C) 1000 mg; pyridoxine (vitamin B6) 6.5 mg; cyanocobalamin (vitamin B12) 9.6 μg; folic acid 400 μg; iron 5 mg; zinc 10 mg; selenium 110 μg; copper 0.9 mg; and contained the same excipients as placebo.

Randomization was accomplished with a prior randomization list, which assigned each patient to one arm or the other in the order listed. To ensure masking, a list was created with the treatment codes assigned to each patient in order of inclusion. This code was registered in the electronic case report form on the page of the baseline visit of each patient, to enable the participant–treatment relationship assigned to be established. Patients were assigned sequentially as they entered the study.

Patients were offered the option to participate in the study, signed the informed consent, and were assigned sequentially as they entered the study, with an allocation ratio of 1:1 (experimental–placebo); in both cases, they received one oral effervescent tablet to be taken daily for 14 days. Subsequent telephone visits were performed every 2 days during the first week, once a week between day 22 and day 30 of the study, and later biweekly between day 30 and day 90. An on-site visit was completed on day 90 to assess persistent symptoms of COVID-19 through a standardized anamnesis and an analytical assessment. A final phone call on D180 (month 6) was conducted to assess quality of life and cognitive status. Randomization lists and treatment blinding were performed by personnel from the hospital pharmacy department not involved in the conduct of the trial. The unblinding procedure was carried out after the last patient’s last visit had been performed.

### 2.6. Outcomes 

The primary outcome was assessment of the need for hospital admission for SARS-CoV-2 infection and the incidence of PCC or long COVID within the 6-month follow-up period, after a 2-week supplementation with an MMS in acute COVID-19 outpatients (since long-term, persistent symptoms can impair quality of life in this population). The study planning is shown in Supplementary Materials (Material S1).

Different secondary outcomes were considered: (i) the micronutritional status prior to the administration of the MMS and at the end of the study; (ii) the effect of the quality/quantity of food intake at the onset of symptoms in the development of PCC; (iii) adverse events reports; (iv) cognitive status assessment at the beginning and end of the study, using the Montreal Cognitive Assessment (MoCA)-BLIND with different cognitive domains: attention and concentration, memory, language, conceptual thinking, calculations, and orientation [16]; and (v) quality of life at the initiation and end of the study, using the EuroQol 5 dimensions 5 levels (EQ-5D-5L) instrument (a self-assessed, health-related quality of life questionnaire) for Spanish populations at the initiation and end of the study [17]. The scale measures quality of life on a five-component scale, including mobility, self-care, usual activities, pain/discomfort, and anxiety/depression. See Supplementary Materials (Material S2).

The MoCA-BLIND test was selected because the final visit at D180 was performed through a telephone call to objectively assess symptoms of neurological impairment, such as attention and memory deficits typical of PCC or neuro-long-COVID [18].

We used duplicate measurements and range checks of outcome data values to ensure the quality of outcome data during data collection.

### 2.7. Treatment-Related Side Effects

As the MMS contained micronutrients at dosages below the upper tolerable limits, we considered it safe for daily use over the long term. Nevertheless, because of the clinical trial design, it was important to register any adverse events, including serious adverse events (SAEs). All adverse events reported by patients, both serious and non-severe, were collected. The instructions for grading seriousness, intensity, and causality are described in the Supplementary Materials (Material S1).

### 2.8. Statistical Analysis

Sample size calculation used the ARCSINUS approach and considered an alpha risk of 0.05 and a beta risk of <0.2 in a bilateral contrast, estimating a follow-up loss rate of 20% with the POISSON approach. For the sample size calculation, we considered a 30% incidence for PCC and 0.5 reduction in relative risk (RR), assuming a 0.37 for the non-exposed group. The endpoints were evaluated in the intention-to-treat (ITT) population. Comparison between the two groups used the logistic regression model adjusted to cofactors. A logistic regression model was used to calculate the odds ratio (OR) and 95% confidence intervals (CI) for the probability of developing PCC, using the placebo group as the reference group. All analyses were performed with the R statistical package. Results are presented as percentages, mean and standard deviations, or median and interquartile range (IQR).

## 3. Results

The results reported in this trial have been written following the CONSORT 2010 statement [19]. 

### 3.1. Study Participants

**Figure 1:**
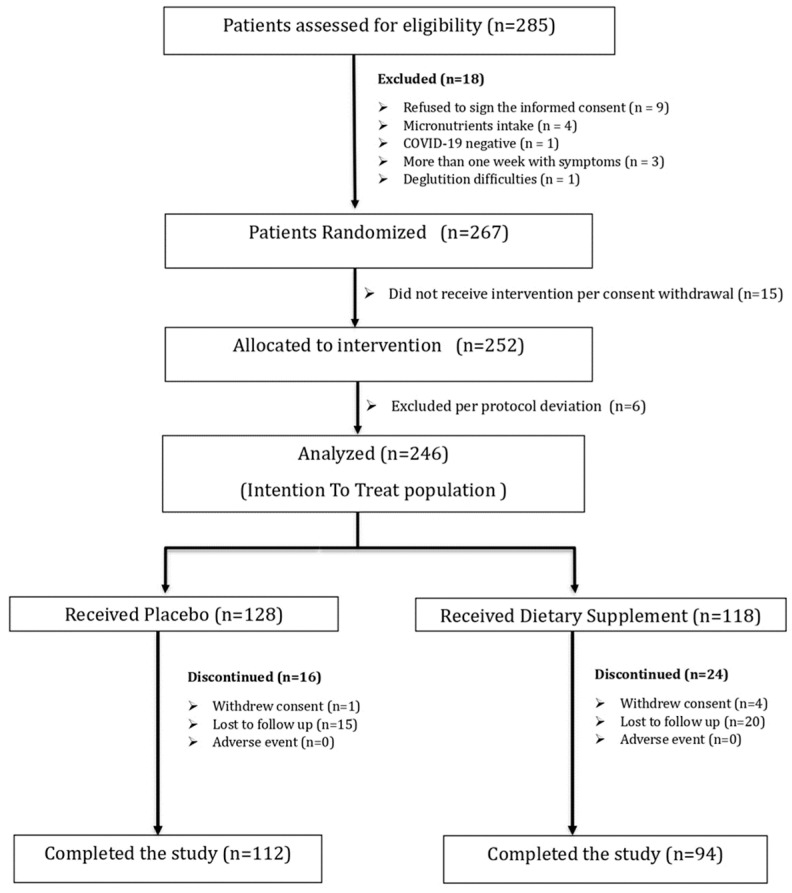
Flow-chart. Enrolment and randomization.

Among the 285 patients assessed for eligibility, 267 underwent randomization. Fifteen patients withdrew consent before receiving the MMS and six were considered a protocol deviation. The required sample size was achieved, with 246 subjects included in the ITT population. Overall, 118 patients were assigned to the MMS group and 128 to the placebo group. Forty patients discontinued during the study period (Figure 1).

### 3.2. Recruitment 

Patients were recruited from the emergency department when they went for consultation due to COVID-19 symptoms, and from the occupational risk department of a tertiary hospital when they started a sick leave because of acute COVID-19 disease.

### 3.3. Baseline Data 

The mean age of the patients was 46.8 years, 68.3% were female, and the mean BMI was 25.7 kg/m^2^. Overall, 91% of patients were white. The main coexisting conditions were hypertension (18.3%), dyslipidemia (13.8%), and obesity (12.2%). A previous COVID-19 episode was reported in 31.7% of patients, and 93% had been vaccinated against SARS-CoV-2. The current COVID-19 episode occurred an average of 232 days after the third vaccine dose. The demographic and clinical characteristics of the randomized population was well matched between the groups (Table 1). 

The most frequently reported symptoms in the acute phase were fatigue (80.9%), mucus (74.5%), cough (67.5%), headache (60.6%), fever (52.8%), myalgia (52.8%), and odynophagia. (52%). Regarding usual food intake in the week prior to inclusion, 77.3% of patients reported having eaten as usual, 12.8% between 50 and 75% of their usual intake, 6.2% between 25 and 50%, and 3.7% reported having eaten <25% of their usual intake. 

### 3.4. Main Results

The D180 telephone visit was completed in 206 patients (83.7%).

#### 3.4.1. Primary Outcomes

Only three of the 246 patients analyzed in the study required hospital admission due to worsening COVID-19 symptomatology.

The incidence of PCC was 26.2% during the 6-month follow-up (final visit). The most frequently recorded persistent symptoms were neurological (14.1%), followed by neurosensory (10.2%) and respiratory (8.7%). No significant between-group differences were observed among symptoms (respiratory, cardiovascular, digestive, neurological, neurosensory, psychological, and others) (Table 2).

**Figure 2:**
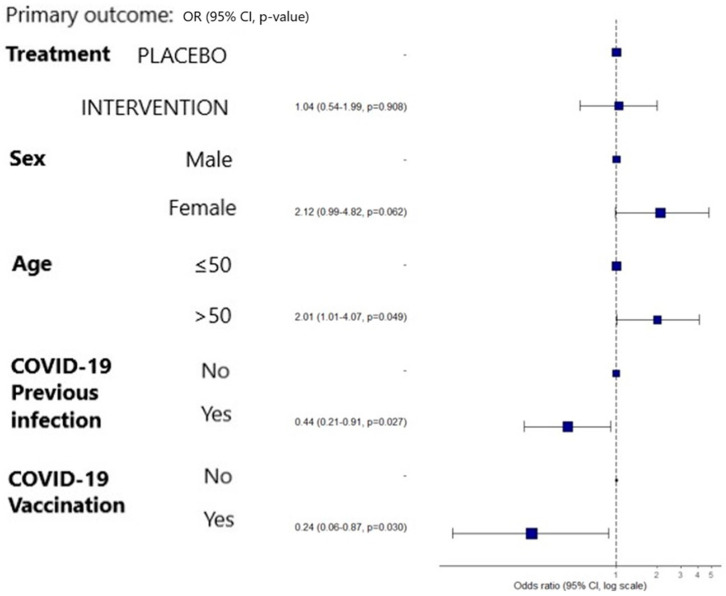
Primary outcome: 6-month PCC-related factors (logistic regression model).

No significant differences were observed in the primary outcome between groups—the percentage of patients with PCC at the D180 follow-up visit was similar between groups: 25.0% in the placebo group and 27.7% in the MMS group (*p* = 0.785; RR = 1.1). Similar results were obtained for the OR values; the logistic regression model revealed that the variables SARS-CoV-2 vaccination [OR 0.2384, 97.5% CI 0.0623–0.8727, *p* = 0.0295] and previous SARS-CoV-2 infection [OR 0.4387, 97.5% CI 0.2093–0.9098, *p* = 0.0272] were PCC protective factors. The main PCC risk factors were age over 50 years (OR 2.0072, 97.5% CI 1.0102–4.0714, *p* = 0.0490) and female sex (OR 2.115, 97.5% CI 0.9855–4.8217, *p* = 0.0624) (Figure 2).

#### 3.4.2. Secondary Outcomes

##### Micronutrient Status

Plasma values of several micronutrients were analyzed twice: at enrollment in 225 (91.5%) patients and at the D90 follow-up visit in 189 patients (84%). Low levels of one or more micronutrients were detected in 54.6% of patients at baseline (Table 3) and in 46% at the D90 follow-up visit.

Based on their baseline micronutritional status, we observed that patients who had micronutritional plasma levels below the lower limit of the reference were older (*p* = 0.044), more likely to be male (*p* = 0.031), had a higher BMI (*p* = 0.008), had more comorbidities (*p* = 0.005), and had a greater intake of common drugs such as oral antidiabetics (*p* = 0.034) and statins (*p* = 0.001) (Table 4). In addition, inflammation seemed to be more marked in patients with micronutritional plasma levels below the lower limit of the reference, since ferritin levels (mean (SD)) trended to be higher than in those with more than one deficit (252.34 ng/mL (215.94) vs. 169.74 ng/mL (268.92), respectively; *p* = 0.060). 

Overall, the MMS was unable to demonstrate any statistically significant clinical benefits to improve micronutrient status at D90 follow-up visit, in patients whose baseline micronutrient levels were below the lower limit of the reference or those within the normal range.

##### Neurological Impairment and Quality of Life

The most common persistent symptoms were neurological, both at 3- and 6-month follow-up visits. Neurological symptoms persisted in 52 of 199 patients evaluated (26.1%) at the D90 follow-up visit and in 28 of 191 patients (14.7%) at the D180 follow-up visit (Table 2). Micronutritional plasma levels at baseline were below the lower limit of the reference in 18.3% of patients with persistent neurological symptoms, while 10.3% had normal micronutrient status (*p* = 0.302). Concerning memory loss symptoms recorded at D180, up to 14.9% had micronutritional plasma levels below normal at baseline, whereas only 4.6% had normal micronutrient status (*p* = 0.038). 

Both the worse quantitative/qualitative food intake (OR 3.3, 97.5% CI 0.8–13.5, *p* = 0.082) and the poorer micronutrient status at the onset of symptoms (>1 deficit) (OR 1.8, 97.5% CI 0.8–4.2, *p* = 0.171) were non-significant, but trend risk factors for PCC. Additional results are available in the Supplementary Materials, Table S1.

To assess cognitive status, the MoCA-BLIND test was performed in a subset of patients after modification of the study protocol. At the D180 follow-up visit in 91 patients, there was a worsening test score in 22 patients (24.2%) compared to baseline. In this subgroup of patients, we observed even worse MoCA-BLIND test results in the placebo group (16 patients (30.8%)) compared to the MMS group (six patients (15.4%)). As observed for the poorer micronutritional status, a tendency (*p* = 0.233) was also found for less cognitive impairment in the MoCA-BLIND test in those who received the MMS. However, only 37% of the sample was available for this analysis and it was not statistically significant (Table 5).

In the quality-of-life analysis (EQ-5D-5L), no statistical differences were found between the treatment groups. The analysis was performed in 142 patients at baseline (mean score 5.77 (1.11)) and at D180 (mean score 5.67 (1.16)). Additional results are available in the Supplementary Materials, Table S2. 

##### Safety Outcomes

In the ITT population, adverse events occurred in 195 out of 246 patients (79.3%) and were slightly more numerous in the MMS group (98 patients (83.1%)) than in the placebo group (97 (75.8%)) (*p* = 0.001), including four pregnancies. The intensity of the adverse effects was mild in 89.5%, moderate in 9.1%, and severe in 1.4% of patients, with no differences detected between the MMS and placebo groups (*p* = 0.711). Adverse effects were not treatment related in 85.8%, with no differences between groups. Conversely, adverse events were documented as probable/definite adverse events in 7%, with more reported in the MMS than placebo group (3.2% vs. 10.6%, respectively, *p* < 0.001). A probable or definite adverse event was defined as follows: the temporal relationship between the investigational product and the adverse event indicates a possible causal relationship and cannot be explained by factors such as the patient clinical status, or other therapeutic interventions. These side effects were mild, and the MMS did not result in any adverse events other than a change in urine color (a well-known and harmless consequence of vitamin B supplementation).

Concerning SAEs, neither of the two SAES reported was treatment related. No deaths were recorded. Two patients required hospitalization, one because of a urinary tract infection and the other one for acute diverticulitis; these patients had been assigned to the MMS and placebo groups, respectively. No hospitalizations were labeled as treatment related.

Regarding the outcome of adverse events, 81.3% of patients had complete recovery, with no differences between groups. The rate of non-recovery of the event was identical between the groups (14.8%). The adverse event led to sick leave in 6.8% of cases (19 (7.6%) in the placebo group and 16 (6.0%) in the MMS group), without significant differences. Finally, the need for medication to treat the adverse effect was 66.8% in the placebo group vs. 61.4% in the MMS group (*p* = 0.233). For those adverse events considered significant, no differences were recorded between groups (11.2% in the placebo group and 7.2% in the MMS group). Additional results are available in Supplementary Materials, Table S3.

## 4. Discussion

Our randomized controlled trial is the first study to evaluate the benefits of multimicronutrient supplementation on the incidence of PCC following a mild, acute COVID-19 episode. The study population was predominantly white, middle-aged women, half of whom had low micronutrient status at baseline. In this population, MMS for 14 days at the onset of symptoms did not improve their clinical outcomes, the incidence of PCC, or their micronutritional status at the 3-month follow-up. 

Unlike our study, other authors did find positive effects of micronutrient treatment on PCC. In a study with 46 adult outpatients with mild COVID-19, supplementation for 28 days with L-arginine plus vitamin C improved walking performance, muscle strength, endothelial function, and fatigue, suggesting this treatment could alleviate persistent symptoms of this disease [20]. 

Currently there is no well-defined description of PCC, despite the UK National Institute for Health and Care Excellence, the World Health Organization, and the US Centers for Disease Control and Prevention publishing their definitions of PCC between December 2020 and October 2021 [21]. We documented a 26.2% incidence of PCC following an acute episode of mild COVID-19—a worrying finding, if transferred to the general population. Other studies have reported similar findings. In a study of 277 adult patients, PCC was detected in 141 patients (50.9%)—34.3% in mild and 65.7% in severe presentations of COVID-19 [22]. In another trial, a PCC incidence of 30–60% was reported, mainly among women, and the risk of persistent symptoms had a linear relationship with age [8]. The main risk factors for PCC, such as age and sex, are not modifiable factors. Female patients have a more pronounced immune response, possibly due to the difference in the expression of sex hormones that may lead to a less severe acute infection, but a greater risk of developing long COVID syndrome [18]. 

In terms of prevention, vaccination was the main protective factor against PCC in our study. Fortunately, this is a potentially modifiable factor to prevent PCC, especially for the most vulnerable at-risk population. Vaccination was also the most relevant factor in other published studies [23,24,25,26]. Up to 18% of people unvaccinated for SARS-CoV-2 before an acute episode have been reported to suffer persistent symptoms two years later [27]. 

Another interesting finding is the high number of patients with persistence of neurological symptoms in our study: 26.1% at D90 and 14.7% at D180. Likewise, the MoCA-BLIND test performed at D180 compared to baseline showed less cognitive impairment in the MMS group, twice as low as placebo; however, the result was not statistically significant (*p* = 0.233), probably due to the small number of patients in both groups. We found that memory loss in PCC could be related to a deficient micronutrient status (*p* = 0.038), and also observed a tendency towards improvement in the MoCA-BLIND test with MMS. Accordingly, it is possible that MMS intake in the acute phase of SARS-CoV-2 might prevent memory loss over 6 months in patients with micronutrient deficits. However, this hypothesis needs further investigation. Other authors have studied 194 patients with mild or severe COVID-19 and also explored the MoCA-BLIND test, finding cognitive impairment in 12.6% of patients [28]. The assessment of cognitive impairment was compared with other groups (patients with COVID-19 without persistent symptoms and patients without COVID-19), but did not use the same evaluation tool in the same group of patients at baseline and at the end of the study. A further study showed that the median MoCA score in COVID-19 patients (26 (25–28)) was significantly lower than that in healthy controls (30 (28.75–30), *p* < 0.001) [29]. In this study, neuro-long-COVID patients with a suboptimal MoCA test exhibited impaired inhibition and dysregulation of the neurotransmitter GABA-b in the glutamatergic system. Therefore, it is possible that neuro-long-COVID could be related to GABA-b inhibitory and glutamatergic facilitatory circuits [29].

There is evidence to support organic and functional brain lesions in the COVID-19 disease. In a study comparing 86 PCC patients with 36 healthy controls undergoing cognitive and neuroimaging examinations 11 months after the acute episode of COVID-19, there was a reduction in brain volume in PCC patients compared to controls. Gray matter volume loss showed significant associations with cognitive dysfunction. These cognitive and brain impairments were more pronounced in hospitalized patients than in outpatients, and no associations were found with vaccination status [30]. Similarly, another study compared organic and functional brain lesions in 401 patients with SARS-CoV-2 infection and in 384 controls [31]. The authors observed a greater reduction in gray matter thickness and tissue damage in regions functionally connected to the primary olfactory cortex in patients with COVID-19, who also showed worse cognitive impairment. Moreno-Pérez et al. identified PCC in 50.9% of patients, with mostly mild symptoms; however, the neurological symptoms (headache, memory disorders/cognitive impairment, or both) were recorded in up to 11.9% of patients [22]. 

Patients with PCC more frequently experienced a negative impact on their quality of life compared to those without sequelae (66.9% vs. 43.2%, respectively, *p* = 0.0001) [22]. In our study, a negative impact on quality of life was also observed at the 6-month follow-up, quantified (EQ-5D-5L) in 22.5% of the 142 patients with mild COVID-19 tested at baseline and at the end of the study. Finally, a survey of 14,767 participants at least 2 months after an acute episode of COVID-19 suggests that cognitive symptoms are common, mostly referred to as mental confusion, associated with worse quality of life [32].

Fifty percent of patients had micronutritional plasma levels below the lower limit of the reference at baseline in our study. Vitamin D was most commonly under the range in our population, followed by vitamin C, then vitamin A. In addition, some B vitamins were below the range, such as B6 and B9. Selenium was the most representative trace element under range. The most common micronutrient deficiencies in the population, in addition to iron and iodine, are vitamin A, folate, and zinc deficiencies [33]. Hypovitaminosis D increases the risk for viral respiratory infections; for this reason, preventive treatment with vitamin D has been proposed to minimize the number and severity of viral infections [34]. However, the administration of vitamin D did not prevent SARS-CoV-2 infection in the CORONAVIT Trial, nor did it seem to affect long-COVID symptoms at the 6-month follow-up period [35]. In our study, patients who had micronutritional plasma levels below the lower limit of the reference had a higher baseline inflammation status, were older, suffered from more medical conditions, and consumed polypharmacy. We are uncertain whether inflammation due to the COVID-19 disease caused the lower micronutritional plasma levels, or whether they were due to coexisting factors of the patients. Inflammation, thrombosis, an autoimmune response, or a persistent viral load appear to be the main contributors to PCC [36]. Oxidative stress and inflammation are implicated in the development and progression of fatigue and neuropsychiatric symptoms. The use of intravenous vitamin C can improve oxygenation and inflammatory markers and appears to reduce the risks of severe courses of the disease. Vollbracht et al. described how intravenous vitamin C in acute COVID-19 could reduce the risk of severe courses and the development of long COVID [37]. Another possibility might be that concomitant medication could cause micronutritional deficiencies [38]. The effects of commonly used drugs on micronutrient homeostasis may be relevant. We found that patients taking oral antidiabetics and statins chronically had significantly worse micronutrient deficiencies than those who did not use them. Oral antidiabetics such as metformin can decrease vitamin B12 levels, while statins decrease vitamin D levels. Other commonly used medications may also reduce micronutrient levels, such as angiotensin-converting enzyme inhibitors (zinc), calcium channel blockers (potassium), thiazides (zinc and B vitamins), loop diuretics (zinc, vitamins B1 and B6), selective serotonin reuptake inhibitors (SSRIs; folic acid), and tricyclic antidepressants (vitamin B2) [39]. 

Vaccination is certainly the best preventive defense against PCC, and it becomes even more important in high-risk populations, such as immunosuppressed patients, those aged over 50 years of age, and women in general. Vaccination against COVID-19 before acute SARS-CoV-2 infection is associated with a lower risk of PCC; however, it is not guaranteed that vaccination will improve symptoms in already ongoing PCC [40]. 

Around 65 and 400 million people across the globe have sought medical advice for PCC [41,42]. As 12–24% may develop neurological impairments, it is imperative to promote research strategies to elucidate the pathophysiological conditions and more personalized treatments for cognitive dysfunction after SARS-CoV-2 infection [43]. Four clinical phenotypes have been identified, one of which resembles chronic fatigue syndrome, with headache and memory impairment [44]. The water-soluble vitamins (B group and C) and zinc are the most relevant micronutrients with an impact on cognitive impairment [45]. Likewise, exposure to multiple essential trace elements may be linked to a protective mechanism against cognitive impairment in older adults [46]. Low serotonin levels may also be key in the cognitive function of PCC in an experimental model [47]. Serotonin is one of the key neurotransmitters in neuronal functions, and SSRIs block the reabsorption of serotonin into neurons; almost 10% of patients in our study were under SSRI treatment. Consequently, increasing serotonin levels in patients with PCC to improve neurological impairment could be an interesting issue to study. Since group B vitamins mediate the conversion of tryptophan into serotonin, analyzing whether an MMS could minimize the number and severity of these persistent neurological symptoms may be of interest.

### Limitations

Once the fifth wave of the pandemic had passed in Spain, COVID-19 cases drastically decreased; the subsequent very slow recruitment rate prolonged the inclusion period for 2 years. Moreover, the incidence of hospital admission was substantially lower during that period, especially because of the increase in the vaccinated population; this made it impossible to have a large enough sample to analyze some of our study objectives. Ultimately, the study reached the required sample size, despite the declining interest in participating in a mild COVID-19 study because people had less concern about the disease and its consequences. Moreover, the majority of patients completed the visits at 3 (88.4%) and 6 months (84.9%). Due to the slow recruitment, we redirected the search towards healthcare workers who were more motivated to participate as patients in the study than the general population. This may be why the population was predominantly women (68.3%) of working age (46.8 years).

Regarding the MMS, both dosage and duration were arbitrary and debatable. We chose an MMS that provided the daily needs of micronutrients for an intake of 2 weeks. However, this could have been insufficient to cover the micronutritional metabolic needs of patients in the event of illness, which would presumably be greater. This is why we also analyzed patients without micronutrient deficiencies at baseline. However, we were unable to find a lower incidence of PCC in MMS patients compared with placebo patients (52.4% vs. 47.6%, *p* = 0.592) (15.1% missing data). The arbitrarily delivered administration period of 2 weeks is also questionable. The idea was to provide an adequate micronutritional status during acute infection, a period in which the patient could have consumed less or poorer nutritional quality food, which we estimated could have been 14 days from the onset of symptoms. It did not seem appropriate to provide MMS at high doses or for prolonged periods in patients with mild COVID-19, but it is possible that both the dose and timing may not have been sufficient to produce any benefit to prevent PCC.

## 5. Conclusions

Our randomized controlled trial in patients with mild, acute COVID-19 found that MMS dispensed at the onset of symptoms for 14 days did not improve the micronutritional status 3 months later and had no impact on the incidence of PCC throughout the 6-month follow-up. Half of the patients had low levels of micronutrient at baseline and a quarter had persistent symptoms at a D180 follow-up visit. Neurological symptoms were the most frequently recorded, and memory loss was more frequently documented in patients with micronutrient deficits than in those with a normal micronutrient status. Our results suggest that the most important risk factors to develop PCC were age over 50 years and female sex, while SARS-CoV-2 vaccination was the most protective.

## Figures and Tables

**Table 1:** General baseline characteristics and medical conditions of the patients.

	Level	Overall	Placebo Group	MMS Group	Missing
n		246	128	118	
Age (mean (SD))		46.83 (16.32)	46.83 (16.36)	46.83 (16.36)	0
Height (mean (SD))		165.90 (8.65)	167.03 (9.26)	167.03 (7.81)	1.2
Weight (mean (SD))		71.13 (15.84)	69.72 (17.00)	72.65 (14.39)	1.2
BMI (mean (SD))		25.73 (4.85)	25.51 (5.11)	25.98 (4.57)	1.2
Sex (%)	Male	78 (31.7)	41 (32.0)	37 (31.4)	0
	Female	168 (68.3)	87 (68.0)	81 (68.6)	
Race (%)	White	223 (91.0)	117 (91.4)	106 (90.6)	0.4
	Black	1 (0.4)	0 (0.0)	1 (0.9)	
	Hispanic	14 (5.7)	8 (6.2)	6 (5.1)	
	Asian	1 (0.4)	1 (0.8)	0 (0.0)	
	Other	6 (2.4)	2 (1.6)	4 (3.4)	
Smoking (%)	Smoker	60 (24.8)	34 (27.2)	26 (22.2)	1.6
	Former smoker ^a^	51 (21.1)	28 (22.4)	23 (19.7)	
	Never smoker	131 (54.1)	63 (50.4)	68 (58.1)	
Drinking (%)	Abstemious	111 (45.9)	55 (44.0)	56 (47.9)	1.6
	Low	122 (50.4)	64 (51.2)	58 (49.6)	
	High	9 (3.7)	6 (4.8)	3 (2.6)	
Chronic health conditions (%)					0
	Diabetes mellitus	20 (8.1)	11 (8.6)	9 (7.6)	
	Hypertension	45 (18.3)	22 (17.2)	23 (19.5)	
	Obesity	30 (12.2)	14 (10.9)	16 (13.6)	
	Dyslipidemia	34 (13.8)	20 (15.6)	14 (11.9)	
Respiratory conditions (%)					0
	COPD	6 (2.4)	2 (1.6)	4 (3.4)	
	Asthma	21 (8.5)	12 (9.4)	9 (7.6)	
	Chronic bronchitis	3 (1.2)	2 (1.6)	1 (0.8)	
	Other	10 (4.1)	4 (3.1)	6 (5.1)	
Cardiological (%)					0
	Arrhythmia	7 (2.8)	4 (3.1)	3 (2.5)	
	Heart failure	1 (0.4)	1 (0.8)	0 (0.0)	
	Valve disease	2 (0.8)	1 (0.8)	1 (0.8)	
	Ischemic ^b^	2 (0.8)	2 (1.6)	0 (0.0)	
	Other (%)	13 (5.3)	6 (4.7)	7 (5.9)	
Gastrointestinal (%)					0
	CAAG	3 (1.2)	2 (1.6)	1 (0.8)	
	GI surgery	3 (1.2)	2 (1.6)	1 (0.8)	
	IBD	1 (0.4)	0 (0.0)	1 (0.8)	
	Other	22 (8.9)	9 (7.0)	13 (11.0)	
Previous COVID-19 episode (%)					1.2
	Yes	77 (31.7)	38 (30.2)	39 (33.3)	
	No	166 (68.3)	88 (69.8)	78 (66.7)	
SARS-CoV-2 vaccine (%)					0
	Yes	227 (93.0)	120 (95.2)	107 (90.7)	
	No	17 (7.0)	6 (4.8)	11 (9.3)	
Type of vaccine (%)					19.5
	Pfizer/BioNTech	150 (75.8)	78 (70.9)	72 (81.8)	
	Moderna	26 (13.1)	14 (12.7)	12 (13.6)	
	AstraZeneca	20 (10.1)	17 (15.5)	3 (3.4)	
	Johnson & Johnson	2 (1.0)	1 (0.9)	1 (1.1)	
Vaccine and boosters (%)					0
	1st dose	225 (91.5)	119 (93.0)	106 (89.8)	
	2nd dose	221 (89.8)	115 (89.8)	106 (89.8)	
	3rd dose	163 (66.3)	80 (62.5)	83 (70.3)	
	4th dose	18 (7.3)	7 (5.5)	11 (9.3)	
Time in days from vaccination and booster to the actual episode of COVID-19 (mean (SD))					
	Since 1st dose	465.55 (178.16)	452.42 (172.06)	480.18 (184.42)	8.9
	Since 2nd dose	438.74 (175.73)	427.84 (160.55)	450.45 (190.80)	10.6
	Since 3rd dose	232.24 (126.15)	225.87 (112.28)	238.30 (138.48)	34.1

^a^ Former smoker (>1 year); ^b^ ischemic heart disease. Abbreviations: BMI: Body Mass Index; CAAG: chronic autoimmune atrophic gastritis; COPD: chronic obstructive pulmonary disease; GI: gastrointestinal; IBD: inflammatory bowel disease; MMS: multiple micronutrient supplement; SD: standard deviation. Missing: missing data in %.

**Table 2:** Patients with persistent symptoms recorded at D180 visit (no missing data).

	Symptoms	Overall	Placebo Group	MMS Group	*p* Value
n		206	112	94	
Patients with persistent symptoms (%)		54 (26.2)	28 (25.0)	26 (27.7)	0.785
Patients with respiratory symptoms (%)		18 (8.7)	11 (9.8)	7 (7.4)	0.724
	Dyspnea	12	8	4	
	Hoarseness	1	0	1	
	Cough	2	2	0	
	Rhinitis	1	1	0	
	Mucus	4	2	2	
Patients with cardiovascular symptoms (%)		2 (0.97)	0	2 (22.2)	0.402
	Palpitations	2	0	2 (100)	
Patients with digestive symptoms (%)		5 (2.4)	3 (2.7)	2 (2.1)	1.000
	Diarrhea	2	2	0	
	Constipation	2	0	2	
	Anorexia	1	1	0	
	Abdominal pain	1	1	0	
Patients with psychological symptoms (%)		4 (1.9)	4 (3.6)	0	0.742
	Apathy	1	0	1	
	Anxiety	5	5	0	
Patients with neurological symptoms (%)		29 (14.1)	14 (12.5)	15 (16.0)	0.610
	Memory loss	16	8	8	
	Ataxia	2	1	1	
	Confusion	1	1	0	
	Headache	6	2	4	
	Insomnia	3	1	2	
	Lack of focus	2	1	1	
	Mental fog	3	1	2	
Patients with neurosensory symptoms (%)		21 (10.2)	7 (6.2)	14 (14.9)	0.070
	Fatigue	18	5	13	
	Pain	7	5	2	
	Anosmia/dysgeusia	4	2	2	
Patients with other symptoms (%)		13 (6.3)	6 (5.4)	7 (7.4)	0.744
	Alopecia	5	2	3	
	Menstruation disorders	2	1	1	
	Dry skin	1	0	1	
	Somnolence	1	0	1	
	Clueless	1	0	1	
	Claustrophobia	1	1	0	
	Joint pain	3	1	2	

MMS: multiple micronutrient supplement.

**Table 3:** Baseline Micronutritional Status.

	Units	Overall	0 Deficit ^a^	1 Deficit ^a^	>1 Deficit ^a^	Missing
Baseline availability (%)		225 (91.5)	102	64	59	
**Vitamin A**						1.3
(Mean (SD))	mg/dL	0.49 (0.17)	0.51 (0.14)	0.53 (0.18)	0.42 (0.19)	
BLR (%)	(<0.300 mg/dL)	26 (11.7)	0 (0.0)	5 (7.9)	21 (35.6)	
**Vitamin B1**						2.2
(Mean (SD))	ng/mL	64.56 (15.36)	64.51 (13.55)	66.82 (15.63)	62.20 (17.62)	
BLR (%)	(<28.00 ng/mL)	1 (0.5)	0 (0.0)	0 (0.0)	1 (1.7)	
**Vitamin B6**						2.7
(Mean (SD))	ng/mL	9.63 (6.10)	10.91 (5.93)	9.12 (3.92)	7.98 (7.73)	
BLR (%)	(<3.6 ng/mL)	19 (8.7)	0 (0.0)	4 (6.5)	15 (25.9)	
**Vitamin B9**						1.8
(Mean (SD))	ng/mL	7.38 (3.67)	7.76 (3.55)	7.22 (2.84)	6.88 (4.57)	
BLR (%)	(<3.5 ng/mL)	19 (8.6)	0 (0.0)	4 (6.3)	15 (25.9)	
**Vitamin B12**						1.8
(Mean (SD))	pg/mL	448.03 (191.74)	493.47 (188.09)	413.56 (147.27)	407.11 (224.10)	
BLR (%)	(<187 pg/mL)	10 (4.5)	0 (0.0)	1 (1.6)	9 (15.5)	
**Vitamin C**						2.7
(Mean (SD))	mg/dL	0.93 (0.47)	1.12 (0.42)	0.86 (0.41)	0.70 (0.50)	
BLR (%)	(<0.40 mg/dL)	35 (16.0)	0 (0.0)	10 (16.1)	25 (42.4)	
**Vitamin D**						1.3
(Mean (SD))	ng/mL	25.25 (12.22)	30.64 (9.11)	23.49 (14.53)	18.11 (9.73)	
BLR (%)	(<20 ng/mL)	71 (32.0)	0 (0.0)	30 (46.9)	41 (69.5)	
**Vitamin E**						1.3
(Mean (SD))	mg/L	13.09 (3.48)	13.27 (3.43)	14.01 (3.78)	11.81 (2.85)	
BLR (%)	(<5 mg/L)	1 (0.5)	0 (0.0)	0 (0.0)	1 (1.7)	
**Copper**						0.4
(Mean (SD))	μg/dL	130.85 (38.69)	131.14 (35.41)	131.98 (36.90)	129.15 (45.94)	
BLR (%)	(<70 μg/dL)	10 (4.5)	0 (0.0)	2 (3.1)	8 (13.6)	
**Selenium**						1.8
(Mean (SD))	μg/L	85.66 (15.13)	89.50 (14.31)	87.66 (15.96)	77.03 (12.05)	
BLR (%)	(<60 μg/L)	8 (3.6)	0 (0.0)	3 (4.8)	5 (8.5)	
**Zinc**						0.4
(Mean (SD))	μg/dL	84.25 (21.50)	87.60 (17.36)	86.77 (27.12)	75.77 (18.94)	
BLR (%)	(<59 μg/dL)	17 (7.6)	0 (0.0)	5 (7.8)	12 (20.3)	

^a^ 0 deficits: patients without micronutritional plasma levels below the lower limit of the reference; 1 deficit: patients with only one micronutritional plasma level below the lower limit of the reference; >1 deficits: patients with two or more micronutritional plasma levels below the lower limit of the reference. Abbreviations: BLR: Below Low Range (below the lower limit of the reference); SD: standard deviation. Missing: missing data in %.

**Table 4:** Micronutritional status regarding treatment for chronic health conditions.

	Overall	0 Deficits ^a^	1 Deficit ^a^	>1 Deficit ^a^	*p*	Missing
n		225	102	64		
Chronic medication (%)	140 (62.2)	60 (58.8)	44 (68.8)	36 (61.0)	0.428	0.0
Inhaled bronchodilators (%)	19 (8.4)	7 (6.9)	8 (12.5)	4 (6.8)	0.386	0.0
Inhaled corticosteroids (%)	11 (4.9)	3 (2.9)	2 (3.1)	6 (10.2)	0.091	0.0
Oral corticosteroids (%)	6 (2.7)	3 (2.9)	1 (1.6)	2 (3.4)	0.799	0.0
Immunosuppressants ^b^ (%)	3 (1.3)	1 (1.0)	1 (1.6)	1 (1.7)	0.914	0.0
Oral antidiabetic agents (%)	18 (8.0)	3 (2.9)	7 (10.9)	8 (13.6)	0.034	0.0
Insulin (%)	5 (2.2)	0 (0.0)	2 (3.1)	3 (5.1)	0.091	0.0
Diuretics (%)	4 (1.8)	1 (1.0)	0 (0.0)	3 (5.1)	0.073	0.0
ACE inhibitors (%)	26 (11.6)	6 (5.9)	10 (15.6)	10 (16.9)	0.052	0.0
ARBs (%)	11 (4.9)	5 (4.9)	5 (7.8)	1 (1.7)	0.291	0.0
Antihypertensive drugs (%)	19 (8.4)	4 (3.9)	9 (14.1)	6 (10.2)	0.063	0.0
PPIs (%)	34 (15.1)	10 (9.8)	11 (17.2)	13 (22.0)	0.097	0.0
Statins (%)	33 (14.7)	6 (5.9)	17 (26.6)	10 (16.9)	0.001	0.0
NSAIDs (%)	7 (3.1)	4 (3.9)	1 (1.6)	2 (3.4)	0.688	0.0
Anxiolytics (%)	12 (5.3)	6 (5.9)	4 (6.2)	2 (3.4)	0.738	0.0
SSRIs (%)	22 (9.8)	10 (9.8)	6 (9.4)	6 (10.2)	0.989	0.0
Probiotics ^c^ (%)	2 (0.9)	1 (1.0)	1 (1.6)	0 (0.0)	0.648	0.0
Other (%)	111 (49.3)	44 (43.1)	35 (54.7)	32 (54.2)	0.238	0.0

^a^ 0 deficits: patients without micronutritional plasma levels below the lower limit of the reference; 1 deficit: patients with only one micronutritional plasma level below the lower limit of the reference; >1 deficits: patients with two or more micronutritional plasma levels below the lower limit of the reference; ^b^ immunosuppressants agents included biologics; ^c^ probiotics, prebiotics and in combination (synbiotics). Abbreviations: ACE: angiotensin-converting enzyme; ARBs: angiotensin receptor blockers; NSAIDs: non-steroidal anti-inflammatory drugs; PPIs: proton pump inhibitors; SSRIs: selective serotonin reuptake inhibitors and other antidepressants. Missing: missing data in %.

**Table 5:** Cognitive status assessment comparison at the beginning and end (D180) of the study (MoCA-BLIND test ^a^).

	Level	Overall	Placebo Group	MMS Group	*p*	Missing
n		91	52	39		
D1 MoCA-BLIND (mean (SD))		17.59 (3.42)	17.52 (3.74)	17.69 (2.97)	0.812	0.0
D180 MoCA-BLIND (mean (SD))		18.43 (3.25)	18.13 (3.49)	18.82 (2.90)	0.322	0.0
D180-D1 Difference (mean (SD))		0.84 (2.31)	0.62 (2.50)	1.13 (2.02)	0.296	0.0
D180-D1 Difference (%)	Worse	22 (24.2)	16 (30.8)	6 (15.4)	0.233	0.0
	Equal	16 (17.6)	8 (15.4)	8 (20.5)		
	Better	53 (58.2)	28 (53.8)	25 (64.1)		

^a^ The MoCA-BLIND test assesses different cognitive domains: attention and concentration, memory, language, conceptual thinking, calculations, and orientation. Abbreviations: D: day; MoCA: Montreal Cognitive Assessment; SD: standard deviation. Missing: missing data in %.

## Data Availability

The data that support the findings of this study are available from the corresponding author upon reasonable request. The data are not publicly available due to privacy.

## Supplementary Materials

The following supporting information can be downloaded at https://www.mdpi.com/article/10.3390/nu16111631/s1; Material S1. Full Protocol; Material S2: MoCA-Blind and EQ-5D-5L tests; Table S1: Food intake and baseline micronutrient status; Table S2: EQ-5D-5L, Quality-of-Life results; Table S3: Adverse Events results.
