# Supplementary material for: Preventive Intake of a Multiple Micronutrient Supplement during Mild, Acute SARS-CoV-2 Infection to Reduce the Post-Acute COVID-19 Condition: A Double-Blind, Placebo-Controlled, Randomized Clinical Trial
Source: Nutrients. 2024 May 26;16(11):1631. doi: 10.3390/nu16111631 (PMC11174626; doi:10.3390/nu16111631)
Supplement: Supplementary file 1 [file nutrients-16-01631-s001.zip › nutrients-3014586-supplementary.pdf]

Preventive Intake of a Multiple-Micronutrient Supplement during Acute SARS-CoV-2 Mild Infection for Reducing Post-Acute COVID-19 Condition: A Double-Blind, Placebo-Controlled, Randomized Clinical Trial

Teresa Maria Tomasa-Irriguible [1,*], Ramon Monfà [2], Cristina Miranda-Jiménez [3], Rosa Morros[4], Neus Robert [5], Luisa Bordejé-Laguna [6], Sandra Vidal [7], Pere Torán-Monserrat [8] and Ana Maria Barriocanal [9]

[1] Intensive Care Department, Hospital Universitari Germans Trias i Pujol, 08916 Badalona, Spain; ttomasa.germanstrias@gencat.cat
[2] Jordi Gol University Research Institute in Primary Care, 08007 Barcelona, Spain; rmonfa@idiapjgol.info
[3] Jordi Gol University Research Institute in Primary Care, 08007 Barcelona, Spain; cmiranda@idiapjgol.org
[4] Autonomous University of Barcelona (UAB), 08193 Bellaterra, Spain; rmorros.bnm.ics@gencat.cat
[5] Emergency Department, Hospital Universitari Germans Trias i Pujol, 08916 Badalona, Spain; nrobert.germanstrias@gencat.cat
[6] Intensive Care Department, Hospital Universitari Germans Trias i Pujol, 08916 Badalona, Spain; lbordeje.germanstrias@gencat.cat
[7] Germans Trias i Pujol Research Institute (IGTP), 08916 Badalona, Spain; svidal@igtp.cat
[8] North Metropolitan Research Support Unit, Jordi Gol University Research Institute in Primary Care (IDIAP Jordi Gol), Mataró, Spain; ptoran.bnm.ics@gencat.cat
[9] Germans Trias i Pujol Research Institute (IGTP), 08916 Badalona, Spain; ambarriocanal@igtp.cat

* Correspondence: ttomasa.germanstrias@gencat.cat

Contact details for the corresponding author:
Teresa-Maria Tomasa-Irriguible
Germans Trias i Pujol Research Institute (IGTP)
Senior Staff –Intensive Care Department
Hospital Universitari Germans Trias i Pujol (HUGTiP) | Ctra. de Canyet, s/n | 08916 Badalona
Gerència Metropolitana Nord | Institut Català de la Salut
ttomasa.germanstrias@gencat.cat | ttomasa@igtp.cat
Tel.(+ 34) 93 497 89 01 | 93 497 82 00

SUPPLEMENTARY MATERIAL

Material S1: Protocol

STRUCTURE OF THE CLINICAL TRIAL. STUDY PLANNING:

Description of the visits

The study was performed under normal clinical practice conditions and was limited to face-to-face visits that responded to obtaining consent, analytical samples, and treatment dispensation. During the follow-up, in addition to the first formal face-to-face study visit, there were also regular and one-way contact through phone to ensure treatment adherence, to collect symptoms and outcome. There was also a regular and one-way contact through messages on the mobile to remind patients their study participation, and study visits.
For patients who might needed to be admitted to the hospital, there was a follow-up by the study team with a weekly visit till study Day 44 and biweekly (till D 90) study visit of the admitted patients, data collection, blood samples, which were complemented until the resolution. By the moment of hospital admission, a new micronutrient analysis was performed.
So that, the study began (D1) and end (D90) with a face-to-face visit, and several phone contacts with patients for clinical research findings and reminders (via SMS to remind patients the visits) until study treatment ended, to confirm that they were well or with mild symptoms.
Patient follow-up timeframe was 180 days.

VISIT 1:
Baseline, face-to-face visit. Inclusion day (day 1). SARS-CoV-2 rapid test confirms COVID19 and obtained ICF. Blood samples were taken in visit 1. Perform Montreal Cognitive Assessment (MoCA-BLIND) test, and EQ-5D-5L (Quality of life assessment) test.

TELEPHONE VISITS (or on site if hospitalized) during study treatment (day 2-14):
Each two-day (on Monday, Wednesday, and Friday) evaluation of symptomatology and complications of the disease.

WEEKLY TELEPHONE VISITS (or on site if hospitalized) (day >15 +/- between 2 and 7 days of window):
Visits for the evaluation of symptomatology, complications of the disease.

FORTNIGHTLY TELEPHONE (or on site if hospitalized) VISITS (day >30 +/- 7 days of window):
Until the end of the study period, for assessment of possible complications developed during this period, the persistence of symptoms and survival.

Contacts via SMS messaging system (whenever necessary):
To remind patients their participation in the study and the study visits.

FINAL VISIT (D90, +/- 7 days of window):
Final and face-to-face visit to determine other possible complications developed during this period, the need for oxygen therapy and survival. Blood samples were also taken in this final

visit. In addition, alt final visit, patients will be asked for the Post-COVID Syndrome and persistent symptoms: Fatigue, anxiety, joints pain, persistent headache, chest pain, dementia, depression, dyspnoea.

FINAL PHONE CALL VISIT (D180, +/- 7 days of window):
Extension of the study x 3 months to evaluate persistent COVID symptoms (1 telephone call visit). Perform Montreal Cognitive Assessment (MoCA-BLIND) test, and EQ-5D-5L (Quality of life assessment) test.

TIMETABLE

	**Baseline visit**	**Each two days phone visits or On site if patient hospitalized**	**If Hospitalization is required**	**Weekly phone visits or On site if patient hospitalized**		**Biweekly phone visits or On site if patient hospitalized**			**Final face-to-face visit**	**Final phone call Visit**
Day of study (*visit window)	D1	D2 to D14	DH	D22 (+/-3 days)	D30 (+/-7 days)	D44 (+/-7 days)	D60 (+/-7 days)	D74 (+/-7 days)	D90 (+/-7 days)	D180 (+/-7 days)
Visit number	V1	V2 to V14	VH	V15	V16	V17	V18	V19	V20	V21
On site visit	X	-	X	-	-	-	-	-	X	-
Telephonic visit	-	X	-	X	X	X	X	X	Optional	X
Informed consent (x2)	X	-	-	-	-	-	-	-	-	-
Selection criteria	X	-	-	-	-	-	-	-	-	-
Rapid SARS-CoV-2 test	X	-	X	-	-	-	-	-	-	-
Medical history review **	X	-	X	-	X	-	X	-	X	X
Randomization	X	-	-	-	-	-	-	-	-	-
Blood sample for micronutrients determination	X	-	X[&]	-	-	-	-	-	X	-
Blood sample (Biochemistry and haematology) (tests performed under routine care are acceptable)	X		X	X[$]						-
Dispensing study treatment	X	-	-	-	-	-	-	-	-	-
Collection of the study treatment	-	-	-	-	-	-	-	-	X	-
Assessment adherence treatment	-	X	X	-	-	-	-	-	-	-
Assessment safety of treatment	-	X	X	X	X	X	X	X	X	-
Assessment symptomatology^^	-	X	X	X	X	X	X	X	X	X
EQ-5D-5L and MoCA-BLIND Tests	X	-	-	-	-	-	-	-	-	X

GENERAL CONSIDERATIONS

The protocol of the study was reviewed by the research Ethics Committees of the Germans Trias i Pujol University Hospital. All researchers and collaborators agree to attend strictly the Helsinki Declaration with all its amendments and to follow the guidelines of good clinical practice of the ICH (International Conference on the Harmonization of Technical Requirements for the Registration of Pharmaceuticals Products for Human Use).

The objectives and methodology, as well as the possible inconveniences and risks due to the study, were explained to each subject orally and in writing (Information sheet of the topic) before its inclusion.

Informed consent was obtained from all patients upon signing the consent form before initiating any study procedure. They were saved in the researcher's archive.

No genetic testing was done with the plasma samples obtained at the visits.

PARTICIPANT INFORMATION SHEET AND INFORMED CONSENT

Informed consent was sought from all participants in the trial, respecting the autonomy of patients in decision-making. The information sheet was provided to the patients in advance and any doubts they may have will be resolved.

Finally, the informed consent was collected in duplicate, duly signed with the patient's date and details. One copy was kept by the patient and the other will be kept on file.

Patients were duly informed in a timely manner of any information that appears and may significantly influence their willingness to continue participating in the study. This information was communicated and documented using a revised consent form or an addendum to the original consent form.

CONFIDENTIALITY

All information obtained in the study was treated confidentially, in compliance with Organic Law 3/2018, of 5 December, on the protection of personal data and guarantee of digital rights and Regulation (EU) 2016/679 of the European Parliament and the Data Protection Council of 27 April 2016 (RGPD).

In the e-Case Report Form, patients were identified only by a code.

SAFETY EVALUATION and ETHICAL ASPECTS

All adverse events reported by patients, both serious and non-severe, will be collected in the data source documentation (patient clinical history).

An adverse event is any untoward medical occurrence in a clinical study subject irrespective of a causal relationship with the study treatment.

ADVERSE EVENT INTENSITY

To assess Adverse Event Intensity this scale will be followed:

- Mild - Transient symptoms that do not influence performance of the patient's daily activities. Other treatment is not indicated.
- Moderate - Marked symptoms sufficient to make the patient uncomfortable. Moderate influence on performance of the patient's daily activities. Other treatment may be necessary.
- Severe - Symptoms cause considerable discomfort. Substantial influence on patient's daily activities. May be unable to continue the study, and other treatment may be necessary.

A Serious Adverse Event is defined as any untoward medical occurrence that, meets at least 1 of the following serious criteria:

- Results in death
- Is life-threatening
- Requires in-patient hospitalization or prolongation of existing hospitalization
- Results in persistent or significant disability/incapacity
- Is a congenital anomaly/birth defect
- Other medically important serious event following the investigator criteria

Hospitalization due to SARS-CoV-2 infection will be collected in all patients as it is the main variable of the study.

The events that fit de definition of thromboses and pulmonary embolism, and death will be considered as variables of the study. They will not be collected as Serious Adverse Events.

ADVERSE EVENT CAUSALITY

The investigator will use clinical judgment to assess the Causality/ Relatedness between the investigational product and an adverse event in the study. Relatedness means that there are facts or reasons to support a relationship between investigational product and the event. Alternative causes, such as underlying disease(s), concomitant therapy, and other risk factors, as well as the temporal relationship of the event to study treatment administration will be considered and investigated.

To assess causality this scale will be followed:

- Related Adverse Event: the temporal relationship between the investigational product and the adverse event indicates a causal relationship possible, and cannot be explained by factors such as the patient clinical status, or other therapeutic interventions
- Not related Adverse Event: the temporal relationship is unlikely, or other factors (concomitant medication or condition), or therapeutic interventions provide a satisfactory explanation for the adverse event.

ADVERSE DRUG REACTION

An Adverse Drug Reaction is any unintended and harmful reaction attributed to the investigational product, regardless of the administered dose.

The events that will be included in the e-CRF will be those that fits de definition of:

- Serious Adverse Event
- Related Adverse Event
- Serious Adverse Drug Reaction
- Adverse Drug Reaction that leads to study treatment withdrawal

PREGNACY

If a female participant (or the female couple of a male participant) becomes pregnant, a follow-up will be performed until the date of deliver, and the outcome of the pregnancy will be reported to the sponsor’s Pharmacovigilance department.

ETHICAL ASPECTS

GENERAL CONSIDERATIONS

The protocol of the study will be reviewed by the research Ethics Committees of the Germans Trias i Pujol University Hospital. All researchers and collaborators agree to strictly observe the Helsinki Declaration with all its amendments and to follow the guidelines of good clinical practice of the ICH (International Conference on the Harmonization of Technical Requirements for the Registration of Pharmaceuticals Products for Human Use).

The objectives and methodology, as well as the possible inconveniences and risks due to the study, will be explained to each subject orally and in writing (Information sheet of the topic) before its inclusion. They will also be informed about the different treatments, how they will be assigned to the groups and the option to withdraw from the study at any time.

Informed consent will be obtained from all patients upon signing the consent form before initiating any study procedure. They will be saved in the researcher's archive.

No genetic testing will be done with the plasma samples obtained at the visits.

PARTICIPANT INFORMATION SHEET AND INFORMED CONSENT

Informed consent will be sought from all participants in the trial, respecting the autonomy of patients in decision making. The information sheet will be provided to the patients in advance and any doubts they may have will be resolved.

Finally, the informed consent will be collected in duplicate, duly signed with the patient's date and details. One copy will be kept by the patient and the other will be kept on file.

Patients will be duly informed in a timely manner of any information that appears and may significantly influence their willingness to continue participating in the study. This information will be communicated and documented using a revised consent form or an addendum to the original consent form.

CONFIDENTIALITY

All information obtained in the study will be treated confidentially, in compliance with Organic Law 3/2018, of 5 December, on the protection of personal data and guarantee of digital rights and Regulation (EU) 2016/679 of the European Parliament and the Data Protection Council of 27 April 2016 (RGPD).

In the e-Case Report Form, patients will be identified only by a code. The names of the participants will not appear in any publication or communication of the results of the study.

The researcher will keep a list with the name of the participants, date of inclusion and their study code.

INSURANCE POLICY

The study treatment is not a medicine, is a dietary supplement that will already be in the Spanish market for the beginning of the study, so a study insurance is not needed.

Despite this situation, because the study is a clinical trial, an insurance policy will be contracted, that will be updated until the study will end.

TRIAL LIMITATIONS

We are in a pandemic situation. Health care centres are at the limit of their possibilities and starting a clinical trial can be a complication for their job.

Contingency: We will need to recruit two physicians (one for the Emergency Department and the other for the admitted patients) and three nurses (one for the Emergency Department, another for the outpatient's follow-up and the other for the admitted patients) for the study project. The project manager will coordinate all actions to verify that all actions of the protocol have been carried out and verify that there are no deviations from the protocol.

Material S2

MoCA-Blind test. Spanish version

MoCA-Blind assesses different cognitive domains: attention and concentration, memory, language, conceptual thinking, calculations, and orientation.

MONTREAL COGNITIVE ASSESSMENT / MoCA-BLIND
(Evaluación Cognitiva Montreal)/MoCA- Discapacidad visual

Versión Mexicana 7.1 Versión Original

Nombre:
Nivel de estudios:
Sexo:
Fecha de nacimiento:
Fecha:

MEMORIA		ROSTRO	TERCIOPELO	IGLESIA	MARGARITA	ROJO	PUNTOS
Lea la lista de palabras, el paciente debe repetirlas. Haga dos intentos. Recuérdeselas 5 minutos más tarde.	1er. Ensayo						SIN PUNTOS
	2do. Ensayo						

ATENCIÓN

Lea la serie de números (1 número/seg.) El paciente debe repetirla en mismo orden [ ] 2 1 8 5 4
El paciente debe repetir la serie de forma la inversa [ ] 7 4 2 __/2

Lea la serie de letras. El paciente debe dar un golpecito con la mano cada vez que se diga la letra A. No se asignan puntos si ≥ 2 errores.
[ ] F B A C M N A A J K L B A F A K D E A A A J A M O F A A B __/1

Reste de 7 en 7 iniciando en el 100
[ ] 93 [ ] 86 [ ] 79 [ ]72 [ ] 65
4 o 5 sustracciones correctas: **3 puntos**, 2 o 3 correctas: **2 puntos**, 1 correcta: **1 punto**, 0 correctas: **0 puntos** __/3

LENGUAJE

Repetir: Sólo sé que Juan es el único que necesita ayuda hoy [ ]
El gato siempre se escondió debajo de la cama cuando los perros estaban en la habitación [ ] __/2

FLUIDEZ / Decir el mayor número posible de palabras que comiencen por la letra **"P"** en 1 min.
[ ]_______ **(N ≥ 11 palabras)** __/1

ABSTRACCIÓN

Similitud entre p. ej. Plátano – naranja = fruta [ ] tren – bicicleta [ ] regla - reloj __/2

RECUERDO DEMORADO	Debe acordarse de las palabras	ROSTRO	TERCIOPELO	IGLESIA	MARGARITA	ROJO	Puntos por recuerdos SIN PISTAS únicamente	__/5
	SIN PISTAS	[ ]	[ ]	[ ]	[ ]	[ ]		
Opcional	Pista de categoría							
	Pista elección múltiple							

ORIENTACIÓN [ ] Día del mes (fecha) [ ] Mes [ ] Año [ ] Día de la semana [ ] Lugar [ ] Ciudad __/6

Adaptación: L. Ledesma PhD., P. García Psic., J. Salvador PhD

Normal ≥ 18 / 22
www.mocatest.org

TOTAL ___22
Añadir 1 punto si tiene ≤ 12 años de estudios

© Z. Nasreddine MD
Administrado por:_______________________________

<u>MoCA-Blind test</u>. English version

MONTREAL COGNITIVE ASSESSMENT / MoCA-BLIND

Version 7.1 Original Version

Name:
Education:
Sex:
Date of birth:
Date:

MEMORY		FACE	VELVET	CHURCH	DAISY	RED	POINTS
Read list of words, subject must repeat them. Do 2 trials even if 1st trial is successful. Do a recall after 5 minutes.	1st trial						No points
	2nd trial						

	POINTS
**ATTENTION**	
Read list of digits (1 digit/sec.) Subject has to repeat them in the forward order [ ] 2 1 8 5 4 Subject has to repeat them in the backward order [ ] 7 4 2	__/ 2
Read list of letters. The subject must tap with his hand at each letter A. No point if ≥ 2 errors [ ] F B A C M N A A J K L B A F A K D E A A A J A M O F A A B	__/ 1
Serial 7 subtraction starting at 100 [ ] 93 [ ] 86 [ ] 79 [ ] 72 [ ] 65 4 or 5 correct subtractions: **3 pts**, 2 or 3 correct: **2 pts**, 1 correct: **1 pt**, 0 correct: **0 pt**	__/ 3
**LANGUAGE**	
**Repeat:** I only know that John is the one to help today. [ ] The cat always hid under the couch when dogs were in the room. [ ]	__/ 2
**Fluency /** Name maximum number of words in one minute that begin with the letter F. [ ] ______ (N ≥ 11 words)	__/ 1
**ABSTRACTION** **Similarity between** e.g. banana - orange = fruit [ ] train - bicycle [ ] watch - ruler	__/ 2

DELAYED RECALL	Has to recall words **With no cue**	FACE [ ]	VELVET [ ]	CHURCH [ ]	DAISY [ ]	RED [ ]	Points for UNCUED recall only	__/ 5
**Optional**	Category cue							
	Multiple choice cue							
**ORIENTATION**	[ ] Date	[ ] Month	[ ] Year	[ ] Day	[ ] Place	[ ] City		__/ 6

© Z. Nasreddine MD **www.mocatest.org** Normal ≥ 18 / 22 TOTAL __/ 22
Add 1 point if ≤ 12 yr edu

Administered by:______________________________

EQ-5D-5L test

EQ-5D-5L

Health Questionnaire

English version for the UK

Sample

© 2009 EuroQol Research Foundation. EQ-5D™ is a trade mark of the EuroQol Research Foundation. UK (English) v1.2

Under each heading, please tick the ONE box that best describes your health TODAY.

MOBILITY

I have no problems in walking about	❑
I have slight problems in walking about	❑
I have moderate problems in walking about	❑
I have severe problems in walking about	❑
I am unable to walk about	❑

SELF-CARE

I have no problems washing or dressing myself	❑
I have slight problems washing or dressing myself	❑
I have moderate problems washing or dressing myself	❑
I have severe problems washing or dressing myself	❑
I am unable to wash or dress myself	❑

USUAL ACTIVITIES *(e.g. work, study, housework, family or leisure activities)*

I have no problems doing my usual activities	❑
I have slight problems doing my usual activities	❑
I have moderate problems doing my usual activities	❑
I have severe problems doing my usual activities	❑
I am unable to do my usual activities	❑

PAIN / DISCOMFORT

I have no pain or discomfort	❑
I have slight pain or discomfort	❑
I have moderate pain or discomfort	❑
I have severe pain or discomfort	❑
I have extreme pain or discomfort	❑

ANXIETY / DEPRESSION

I am not anxious or depressed	❑
I am slightly anxious or depressed	❑
I am moderately anxious or depressed	❑
I am severely anxious or depressed	❑
I am extremely anxious or depressed	❑

Sample

© 2009 EuroQol Research Foundation. EQ-5D™ is a trade mark of the EuroQol Research Foundation. UK (English) v1.2

- We would like to know how good or bad your health is TODAY.
- This scale is numbered from 0 to 100.
- 100 means the best health you can imagine.
  0 means the worst health you can imagine.
- Please mark an X on the scale to indicate how your health is TODAY.
- Now, write the number you marked on the scale in the box below.

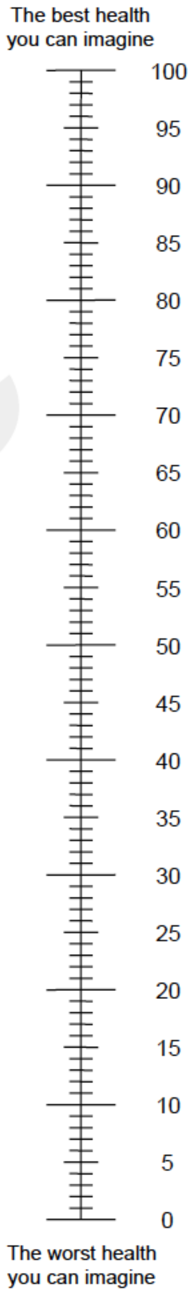

YOUR HEALTH TODAY = ☐

Sample

© 2009 EuroQol Research Foundation. EQ-5D™ is a trade mark of the EuroQol Research Foundation. UK (English) v1.2

Tables S1–S3

Table S1. Food intake in relation to baseline micronutrient status

	level	Overall	0 deficit	1 deficit	≥2 deficits	p	Missing
n		225	102	64	59		
Nutritional status (%) *Variable created from the set of variables in the section	Normal	214 (95.1)	98 (96.1)	63 (98.4)	53 (89.8)		
	Poor	11 (4.9)	4 (3.9)	1 (1.6)	6 (10.2)	0.072	0.0
Is the patient in metabolic stress or is an absence of food intake expected for more than 5 days?	No	220 (99.1)	102 (100.0)	62 (100.0)	56 (96.6)		
	Yes	2 (0.9)	0 (0.0)	0 (0.0)	2 (3.4)	0.058	1.3
Food intake during the last week before inclusion in the study (%)	as usual (≥75%)	170 (76.9)	82 (81.2)	48 (76.2)	40 (70.2)		
	more than half (50-75% of his usual intake)	29 (13.1)	12 (11.9)	11 (17.5)	6 (10.5)		
	less than half (25-50% of your usual intake)	14 (6.3)	5 (5.0)	3 (4.8)	6 (10.5)		
	much less (< 25% of usual intake)	8 (3.6)	2 (2.0)	1 (1.6)	5 (8.8)	0.139	1.8
Legumes (%)	0 days	8 (3.6)	3 (2.9)	2 (3.2)	3 (5.3)		
	1	48 (21.7)	26 (25.5)	11 (17.7)	11 (19.3)		
	2	83 (37.6)	35 (34.3)	31 (50.0)	17 (29.8)		
	3	45 (20.4)	23 (22.5)	10 (16.1)	12 (21.1)		
	4	22 (10.0)	11 (10.8)	4 (6.5)	7 (12.3)		
	5	5 (2.3)	1 (1.0)	1 (1.6)	3 (5.3)		
	6	3 (1.4)	1 (1.0)	1 (1.6)	1 (1.8)		
	7 days	7 (3.2)	2 (2.0)	2 (3.2)	3 (5.3)	0.595	1.8
Meat (%)	0	7 (3.2)	3 (2.9)	1 (1.6)	3 (5.3)		
	1	22 (10.0)	7 (6.9)	9 (14.5)	6 (10.5)		
	2	40 (18.1)	16 (15.7)	13 (21.0)	11 (19.3)		
	3	37 (16.7)	15 (14.7)	10 (16.1)	12 (21.1)		
	4	39 (17.6)	21 (20.6)	10 (16.1)	8 (14.0)		
	5	21 (9.5)	11 (10.8)	4 (6.5)	6 (10.5)		
	6	34 (15.4)	19 (18.6)	9 (14.5)	6 (10.5)		
	7	21 (9.5)	10 (9.8)	6 (9.7)	5 (8.8)	0.854	1.8
Fish (%)	0	28 (12.7)	11 (10.8)	6 (9.7)	11 (19.3)		
	1	61 (27.6)	28 (27.5)	17 (27.4)	16 (28.1)		
	2	60 (27.1)	29 (28.4)	18 (29.0)	13 (22.8)		
	3	48 (21.7)	22 (21.6)	11 (17.7)	15 (26.3)		
	4	8 (3.6)	5 (4.9)	3 (4.8)	0 (0.0)	0.703	1.8

	5	4 (1.8)	1 (1.0)	2 (3.2)	1 (1.8)		
	6	7 (3.2)	3 (2.9)	3 (4.8)	1 (1.8)		
	7	5 (2.3)	3 (2.9)	2 (3.2)	0 (0.0)		
Eggs (%)	0	8 (3.6)	3 (2.9)	2 (3.2)	3 (5.3)		
	1	32 (14.5)	12 (11.8)	8 (12.9)	12 (21.1)		
	2	72 (32.6)	40 (39.2)	16 (25.8)	16 (28.1)		
	3	59 (26.7)	25 (24.5)	19 (30.6)	15 (26.3)		
	4	32 (14.5)	14 (13.7)	13 (21.0)	5 (8.8)		
	5	3 (1.4)	0 (0.0)	1 (1.6)	2 (3.5)		
	6	9 (4.1)	6 (5.9)	1 (1.6)	2 (3.5)		
	7	6 (2.7)	2 (2.0)	2 (3.2)	2 (3.5)	0.396	1.8
Vegetables (%)	0	31 (14.0)	13 (12.7)	10 (16.1)	8 (14.0)		
	1	118 (53.4)	53 (52.0)	31 (50.0)	34 (59.6)		
	2	61 (27.6)	31 (30.4)	18 (29.0)	12 (21.1)		
	3	7 (3.2)	3 (2.9)	3 (4.8)	1 (1.8)		
	4	4 (1.8)	2 (2.0)	0 (0.0)	2 (3.5)		
	5	0 (0.0)	0 (0.0)	0 (0.0)	0 (0.0)	0.748	1.8
Fruit or juice (%)	0	38 (17.2)	22 (21.6)	5 (8.1)	11 (19.3)		
	1	63 (28.5)	23 (22.5)	19 (30.6)	21 (36.8)		
	2	72 (32.6)	34 (33.3)	22 (35.5)	16 (28.1)		
	3	40 (18.1)	20 (19.6)	12 (19.4)	8 (14.0)		
	4	7 (3.2)	3 (2.9)	4 (6.5)	0 (0.0)		
	5	1 (0.5)	0 (0.0)	0 (0.0)	1 (1.8)	0.129	1.8
Cereals (%)	0	17 (7.7)	9 (8.8)	3 (4.8)	5 (8.9)		
	1	116 (52.7)	47 (46.1)	42 (67.7)	27 (48.2)		
	2	58 (26.4)	28 (27.5)	15 (24.2)	15 (26.8)		
	3	27 (12.3)	16 (15.7)	2 (3.2)	9 (16.1)		
	4	2 (0.9)	2 (2.0)	0 (0.0)	0 (0.0)		
	5	0 (0.0)	0 (0.0)	0 (0.0)	0 (0.0)	0.116	2.2
Milk or derivatives (%)	0	24 (10.9)	11 (10.8)	7 (11.3)	6 (10.5)		
	1	81 (36.7)	33 (32.4)	26 (41.9)	22 (38.6)		
	2	76 (34.4)	41 (40.2)	19 (30.6)	16 (28.1)		
	3	29 (13.1)	12 (11.8)	5 (8.1)	12 (21.1)		
	4	11 (5.0)	5 (4.9)	5 (8.1)	1 (1.8)		
	5	0 (0.0)	0 (0.0)	0 (0.0)	0 (0.0)	0.307	1.8

**Table S2. Quality-of-life survey bivariant data.**						
	level	Overall	PLACEBO	INTERVENTION	p	Missing
n		246	128	118		
Result EVA Basal (mean (SD))		73.35 (14.35)	72.97 (14.60)	73.76 (14.14)	0.719	30.5
Result EVA D180 (mean (SD))		76.52 (12.30)	75.41 (12.87)	77.85 (11.52)	0.161	17.9
Disponible valor test D180 & Basal		141 (57.3)	77 (60.2)	64 (54.2)		
Result EVA Basal (mean (SD))		73.62 (13.71)	73.68 (13.55)	73.55 (14.00)	0.956	0.0
Result EVA D180 (mean (SD))		76.75 (12.00)	76.10 (12.32)	77.53 (11.66)	0.484	0.0
Difference EVA D180-Basal (mean (SD))		3.13 (15.73)	2.43 (16.17)	3.98 (15.28)	0.561	0.0
Difference EVA entre D180-Basal (%)	worse	50 (35.5)	28 (36.4)	22 (34.4)	0.431	0.0
	equal	30 (21.3)	19 (24.7)	11 (17.2)		
	better	61 (43.3)	30 (39.0)	31 (48.4)		
Disponible EQ-5D-5L D180 & Basal		142 (57.7)	77 (60.2)	65 (55.1)		
EQ-5D-5L-Result Basal (mean (SD))		5.77 (1.11)	5.87 (1.22)	5.65 (0.96)	0.232	0.0
EQ-5D-5L-Result D180 (mean (SD))		5.67 (1.16)	5.71 (0.92)	5.62 (1.40)	0.614	0.0
Difference D180-Basal (mean (SD))		-0.10 (1.40)	-0.16 (1.32)	-0.03 (1.49)	0.596	0.0
Difference entre D180-Basal (%)	worse	32 (22.5)	19 (24.7)	13 (20.0)	0.691	0.0
	equal	69 (48.6)	35 (45.5)	34 (52.3)		
	better	41 (28.9)	23 (29.9)	18 (27.7)		

Table S3 - Adverse Events						
	level	Overall	PLACEBO	INTERVENTION	p	Missing
n		515	250	265		
Patients with an adverse event (%)	No	51 (20.7)	31 (24.2)	20 (16.9)		0.0
	Yes	195 (79.3)	97 (75.8)	98 (83.1)	0.212	
Number of adverse events/patient (%)	1	61 (31.3)	34 (35.1)	27 (27.6)	<0.001	0.0
	2	51 (26.2)	25 (25.8)	26 (26.5)		
	3	30 (15.4)	13 (13.4)	17 (17.3)		
	4	25 (12.8)	12 (12.4)	13 (13.3)		
	5	17 (8.7)	7 (7.2)	10 (20.2)		
	6	5 (2.6)	2 (2.1)	3 (3.1)		
	7	3 (1.5)	2 (2.1)	1 (1.0)		
	8	1 (0.5)	0 (0.0)	1 (1.0)		
	9	2 (1.0)	2 (2.1)	0 (0.0)		
Intensity (%)	Mild	461 (89.5)	221 (88.4)	240 (90.6)		0.0
	Moderate	47 (9.1)	25 (10.0)	22 (8.3)		
	Severe	7 (1.4)	4 (1.6)	3 (1.1)	0.711	
Causality (%)	Not related	442 (85.8)	216 (86.4)	226 (85.3)		0.0
	Improbable	26 (5.0)	18 (7.2)	8 (3.0)		
	Possible	11 (2.1)	8 (3.2)	3 (1.1)		
	Probable	16 (3.1)	7 (2.8)	9 (3.4)		
	Definitive	20 (3.9)	1 (0.4)	19 (7.2)	<0.001	
Outcome (%)	Recovered	417 (81.3)	201 (80.4)	216 (82.1)		0.4
	Recovered with ER	14 (2.7)	8 (3.2)	6 (2.3)		
	Not Recovered	76 (14.8)	37 (14.8)	39 (14.8)		
	Lethal outcomes	0 (0.0)	0 (0.0)	0 (0.0)		
	unknown	6 (1.2)	4 (1.6)	2 (0.8)	0.749	
Consequences (%)	Sick leave	35 (6.8)	19 (7.6)	16 (6.0)		0.0
	Lethal outcomes	0 (0.0)	0 (0.0)	0 (0.0)		
	Life threatening	1 (0.2)	0 (0.0)	1 (0.4)		
	Hospitalizatio n	2 (0.4)	1 (0.4)	1 (0.4)		
	Disability or invalidity	1 (0.2)	0 (0.0)	1 (0.4)		
	Congenital anomaly	0 (0.0)	0 (0.0)	0 (0.0)		
	Significant event	47 (9.1)	28 (11.2)	19 (7.2)		
	Not serious	450 (87.4)	211 (84.4)	239 (90.2)		
Need for concomitant medication (%)	No	185 (36.0)	83 (33.2)	102 (38.6)		0.2
	Yes	329 (64.0)	167 (66.8)	162 (61.4)	0.233	
